# First person – Hungtang Ko

**DOI:** 10.1242/bio.059234

**Published:** 2022-02-28

## Abstract

First Person is a series of interviews with the first authors of a selection of papers published in Biology Open, helping early-career researchers promote themselves alongside their papers. Hungtang Ko is first author on ‘ Metabolic scaling of fire ants (*Solenopsis invicta*) engaged in collective behaviors’, published in BiO. Hungtang is a PhD Candidate in the lab of David L. Hu at Georgia Institute of Technology, investigating the properties of animal groups as they are involved in collective behaviors.


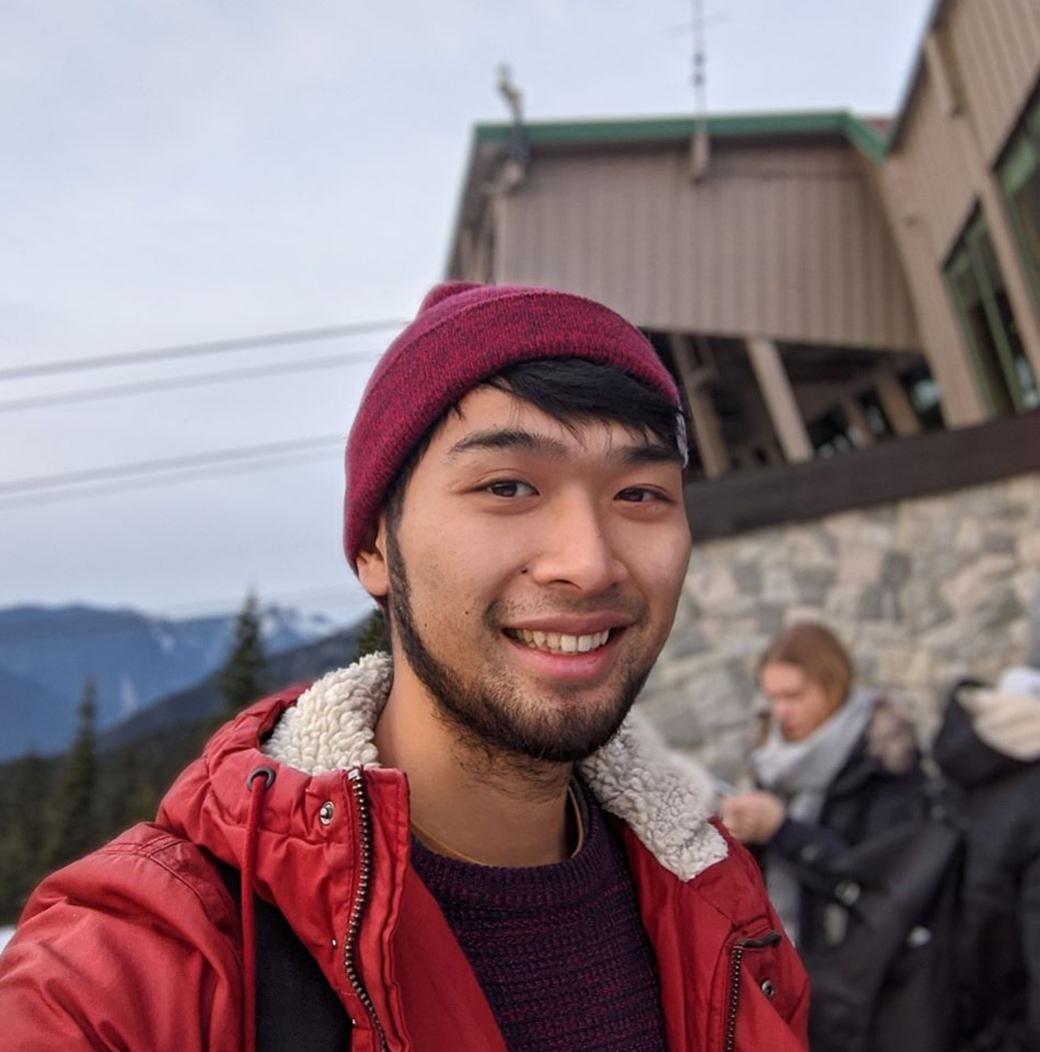


**Hungtang Ko**

**What is your scientific background and the general focus of your lab?**

I majored in theoretical and applied mechanics in my undergrad at Fudan University, China. There I found my passion for fluid mechanics and nonlinear systems. My first research experience with biological organisms occurred in 2017 as I studied *C. elegans* and microfluidic devices at the University of Pennsylvania as a master's student. In my PhD studies at the Georgia Institute of Technology, I further broadened my research interests to include the collective behaviors of insects such as fire ants and black soldier fly larvae. I have also published on the physics of wok tossing with my current advisor Dr David Hu.

All my previous research experience had come together in an unexpected way. In my current field, researchers often compare biological collectives such as fire ant rafts to traditional matters such as fluids and solids. Both types of systems consist of a large number of constituents. The interactions among the constituents give rise to rich and unexpected behaviors of the systems.

**How would you explain the main findings of your paper to non-scientific family and friends?**

Animals become more energy efficient as they grow larger. The metabolic rate per mass decreases with size. In this paper, we investigate if this trend is also true for fire ant groups, especially as they are engaged in tasks that require communication and coordination. While we expect a higher efficiency for larger groups of ants, our measurements revealed that the size effect is absent. This means that ants work as hard in large groups as they do in small groups, a property that we wish student groups could have.

“This means that ants work as hard in large groups as they do in small groups, a property that we wish student groups could have.”

**What are the potential implications of these results for your field of research?**

Our results suggest that animal collectives don't necessarily benefit from size, at least from the metabolic energy consumption perspective. Is this trend the same for other animal groups such as honeybee swarms and mound-building termites? Would larger groups allow them to produce more useful work per individual despite using up the same energy? More research is required to answer these questions.

**Figure:**
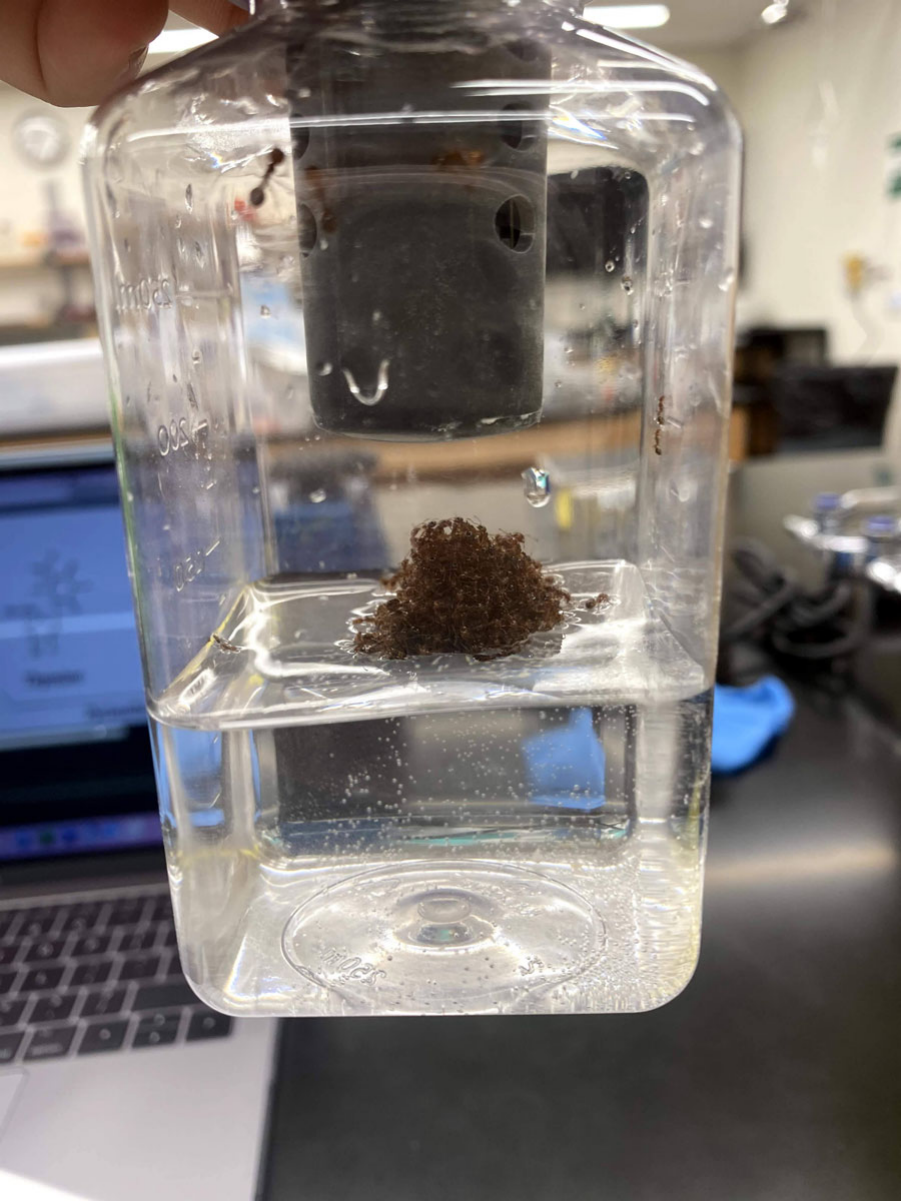
**The carbon dioxide emission rates of fire ant rafts were measured to elucidate how size affects the collectives' metabolism.** Photo credit: Keyana Komilian.

**What has surprised you the most while conducting your research?**

I had thought that ants would spend less energy in large groups like we do!

**What, in your opinion, are some of the greatest achievements in your field and how has this influenced your research?**

In 2010, Hou et al. showed that insect colonies follow allometric scaling for metabolism. This paper helped us form the hypothesis that ants engaged in collective behaviors will also show allometric scaling. Surprisingly, our data rejected the hypothesis.

**What changes do you think could improve the professional lives of early-career scientists?**

I think having more social events would help tremendously. Early-career scientists work by themselves most of the time. Knowing that they belong to a community could benefit their mental health. Cross-disciplinary social events are even better! Connecting with researchers outside of your field can often help people relax. Events like these may also lead to fruitful collaborations.

**What's next for you?**

I expect to graduate in summer 2022 to join a postdoctoral position. I plan to study fish schools! I am interested in how fish interact with the fluid environment and how those interactions affect the properties of fish schools.
